# A facial asymmetry revealed: Active mandibular condylar hyperplasia

**DOI:** 10.1016/j.radcr.2025.01.079

**Published:** 2025-03-08

**Authors:** Asmae Guennouni, Wiam Hormat Aallah, Chaimae Abourak, Siham Oukassem, Najwa Ech-Cherif El Kettani, Meriem Fikri, Firdaous Touarsa, Mohammed Jiddane, Mourad Zekri, Majdouline bellakhdar, Hasnae Guerrouj

**Affiliations:** Neuro-radiology Department, Nuclear Medicine Department, Ibn Sina University Hospital Center, Rabat, Morocco

**Keywords:** Condylar hyperplasia, Mandibular asymmetry, 3D CT, Bone scintigraphy, Condylectomy

## Abstract

Condylar hyperplasia (CH) is a rare condition characterized by excessive growth of the mandibular condyle, leading to facial asymmetry, temporomandibular joint (TMJ) dysfunction, and functional impairments such as chewing and speech difficulties. This report presents a 15-year-old patient with mandibular deviation, limited TMJ mobility, and facial asymmetry. Diagnostic imaging, including CT and planar bone scintigraphy with 99mTc-HMDP, revealed active right condylar hyperplasia, with a 14.29% radiotracer uptake difference, surpassing the 10% threshold for metabolic activity. The patient underwent condylectomy, successfully restoring facial symmetry, masticatory function, and TMJ stability. Early physiotherapy complemented the surgical outcome. This case highlights the importance of combining anatomical and functional imaging modalities for accurate diagnosis and treatment planning. Advanced imaging, such as 3D CT and bone scintigraphy, plays a critical role in confirming condylar hyperplasia activity and guiding surgical decisions. Multidisciplinary management is essential, as timely intervention prevents progression, enhances aesthetics, and improves functionality. Condylectomy remains the treatment of choice for active CH, with orthognathic surgery reserved for severe deformities or malocclusion. Early recognition and comprehensive management of CH are crucial to optimizing patient outcomes and ensuring improved quality of life.

Condylar hyperplasia is a rare condition that affects children and young adults. It is characterized by excessive growth of the mandibular condyle, leading to functional issues such as chewing and speech disorders, as well as aesthetic problems, primarily dominated by facial asymmetry [1].

The condyle is an anatomical region located at the top of each mandible and, with its articular surface, plays a role in the complex movements of the temporomandibular joint. The mandibular condyle also has a significant role in mandibular growth [2].

We report the case of a 15-year-old child presenting with facial asymmetry and temporomandibular joint dysfunction, which led to the diagnosis of condylar hyperplasia.

## Clinical case

**Fig. 1:**
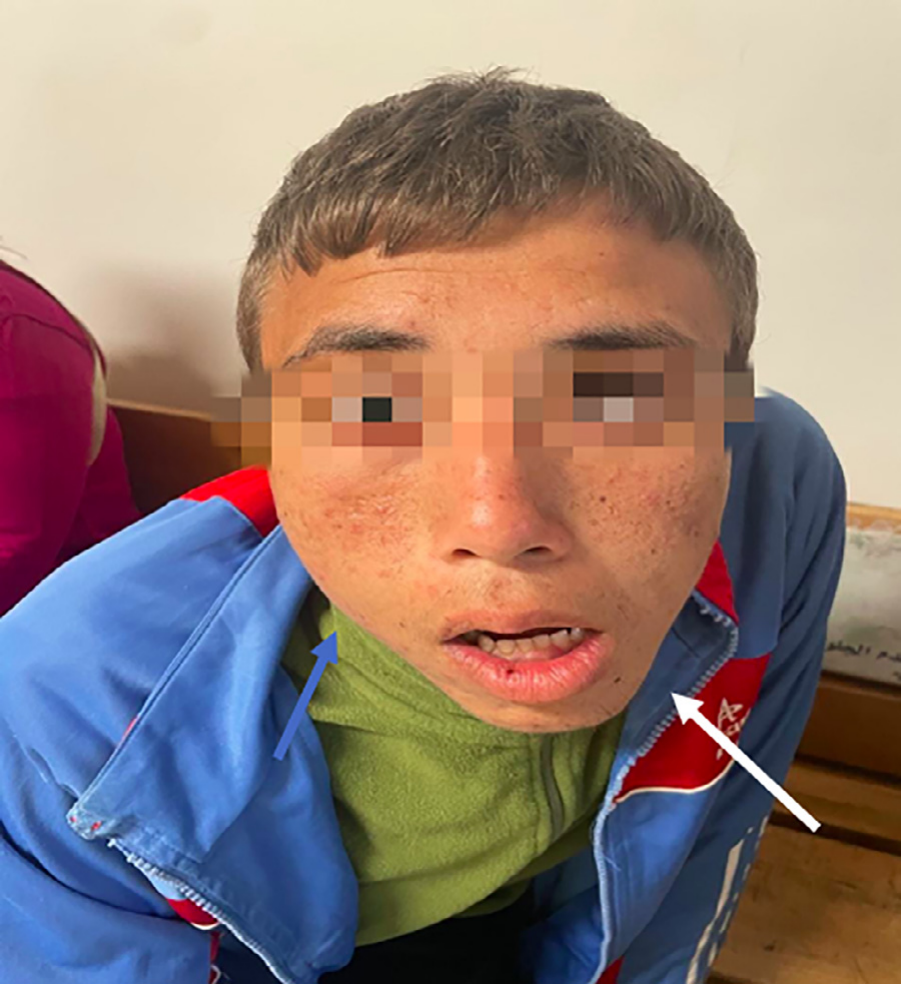
Clinical image of the patient showing facial asymmetry with hypertrophy and lowering of the mandibular angle, as well as an increase in the height of the mandibular ramus (white arrow). A deviation of the chin toward the contralateral side is also observed (blue arrow).

A 15-year-old child with a history of delayed statural and weight development presented with facial asymmetry primarily marked in the mandibular region, with lateral deviation to the right, slight tilting of the maxillary plane, limited temporomandibular joint opening, and instability Fig. 1.

**Fig. 2:**
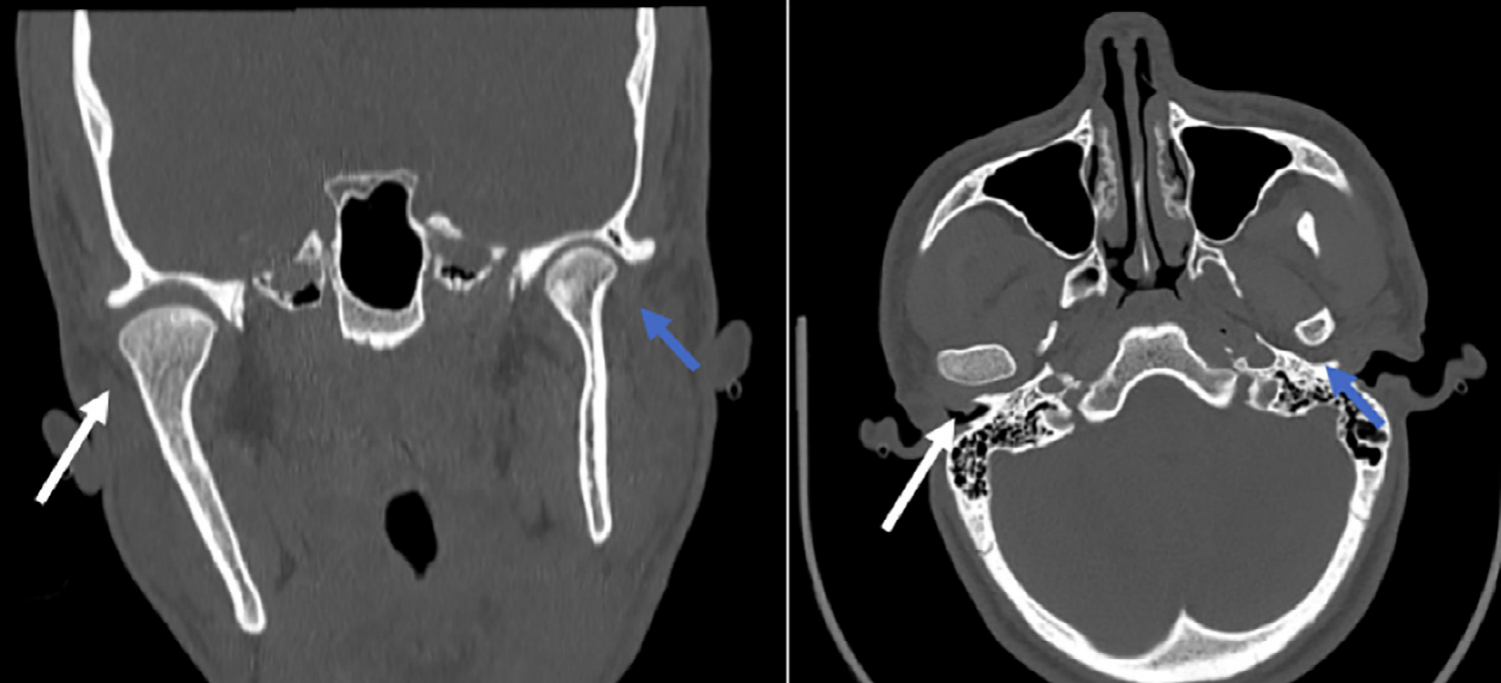
Coronal reconstruction and axial section: right condylar hyperplasia (white arrow) compared to the contralateral side (blue arrow).

**Fig. 3:**
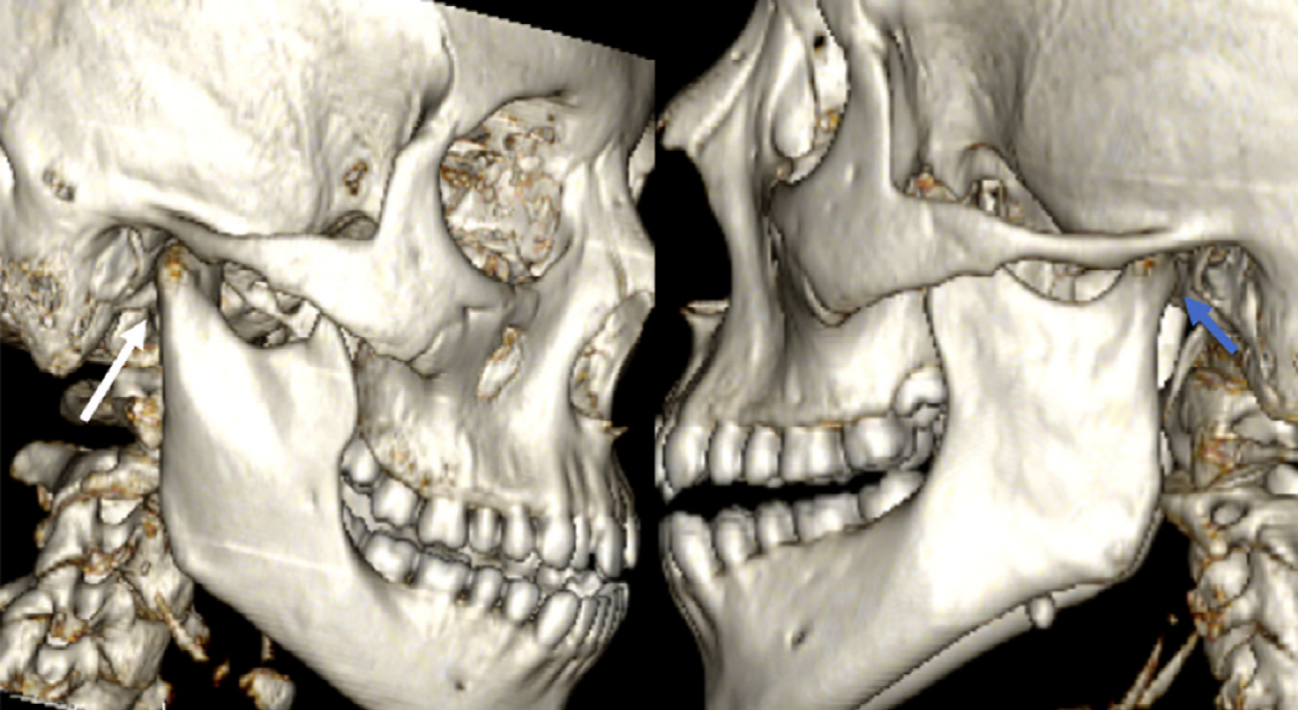
Three dimensional reconstruction showing hypertrophy of the right mandibular condyle (white arrow) compared to the contralateral side (blue arrow).

**Fig. 4:**
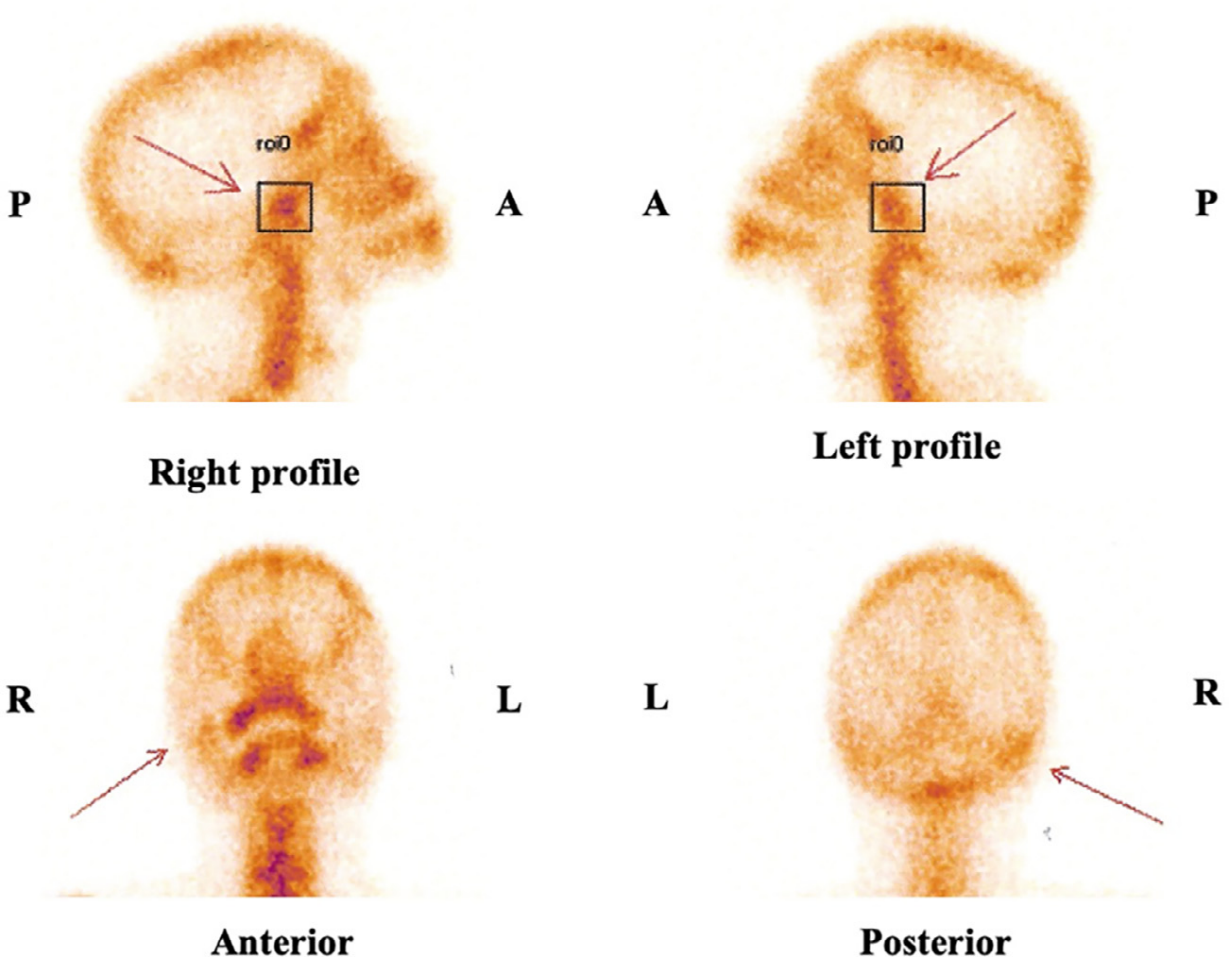
Quantitative planar bone scintigraphy using ^99m^Tc-HMDP, focused on the skull, face, and lateral profile. ROIs placed over the condyles revealed an average radiotracer uptake of 32 counts on the right condyle and 28 counts on the left condyle, yielding a percentage difference in uptake of 14.29%, consistent with mildly active hyperplasia of the right mandibular condyle.

A CT scan was performed, revealing mandibular ramus hypertrophy with incongruence of the condyle in the temporal fossa (Fig. 2, Fig. 3). A planar bone scintigraphy was performed 2 h after intravenous administration of 300 MBq of technetium-99m hydroxymethylene diphosphonate (^99m^Tc-HMDP), with acquisitions focused on the skull, face, and lateral profile. The acquisition protocol included static images acquired over 5 min per view using a high-resolution low-energy collimator. The radiotracer dose was adjusted within the upper range of pediatric dosing recommendations while considering the patient's age and nearing adult weight range, to ensure adequate image quality for diagnostic accuracy. Regions of interest (ROIs) placed over the condyles revealed an average radiotracer uptake of 32 counts on the right condyle and 28 counts on the left condyle. Background counts were analyzed, yielding a standard deviation of 1.2 counts, which ensured the statistical significance of the observed uptake difference between the condyles. The calculated percentage difference in uptake was 14.29%, exceeding the 10% threshold associated with increased metabolic activity. These findings were consistent with mildly active hyperplasia of the right mandibular condyle Fig. 4.

The patient was managed by the maxillofacial surgery department. A total resection of the hypertrophic condyle was performed to halt the progression of facial asymmetry as well as the associated functional complications. This procedure was guided by scintigraphy findings and conducted with meticulous surgical planning.

The intervention successfully restored facial harmony and satisfactory masticatory function, along with proper mouth closure. Postoperative follow-up was established to monitor the stability of the temporomandibular joint and functional adaptation. Physiotherapy was also initiated to optimize mandibular mobility.

## Discussion

Mandibular condylar hyperplasia is defined by the persistence of condylar growth after the cessation of normal growth. It is a rare, benign pathology and one of the most common condylar anomalies.

The development of the mandible, both horizontally and vertically, originates at the condyle and occurs bilaterally, like the rest of the facial skeleton, before the age of 15 for girls and 17 for boys, with a marked acceleration during puberty. Increased condylar activity even after this age is believed to cause hypercondylia [3]. The age of onset is variable, usually between 10 and 30 years, but it may start later, with a female predominance of 69.6% [4].

The etiology is often unknown, but it is generally accepted that hypercondylia results from hyperactivity of condylar cartilage growth. It should be suspected in cases of gradually developing facial asymmetry affecting the lower face, possibly starting as early as 10 years old. It is characterized by the lowering of the labial commissure on the hypercondylia side compared to the contralateral side, hypertrophy with lowering of the mandibular angle, deviation of the chin toward the contralateral side, and increased height of the mandibular ramus.

Panoramic radiography is often the first imaging examination performed when hypercondylia is suspected. It allows visualization of the teeth, dental arches, and mandibular bony structures. Hypercondylia is suspected when asymmetry or hypertrophy of the condyle is present on one side compared to the contralateral side, though it provides little detail on condylar growth or specific temporomandibular joint (TMJ) anomalies.

CT scans are essential for diagnosis, assessing the severity of the asymmetry compared to the contralateral side, evaluating mandibular condyle volume, and planning surgical treatment. They also allow visualization of the enlarged condyle, often asymmetric to the opposite side. Three dimensional reconstructions offer highly precise evaluations of the deformation's dimensions and extent, which are particularly helpful in planning potential surgery [5]. Coronal and axial sections show deviation of the mandible toward the contralateral side of the hypercondylia, leading to facial asymmetry. This deviation has an aesthetic impact due to the increased size of the mandibular region, and a functional impact, potentially affecting mastication and dental occlusion [6].

Bone scintigraphy, typically using ^99m^Tc-HMDP, serves as a functional adjunct in assessing osteoblastic activity in cases of MCH. Although planar scintigraphy has limited spatial resolution, which reduces its precision in localizing uptake within specific condylar regions, it remains effective in identifying asymmetrical tracer accumulation. A difference of over 10% in uptake between condyles is widely considered indicative of active disease [7]. This functional information complements the anatomical findings provided by CT, offering a more comprehensive assessment of condylar growth activity.

Advancements in nuclear imaging have led to the development of SPECT/CT, which combines metabolic and anatomical imaging into a single modality, enhancing both sensitivity and specificity in detecting active MCH. It enables detailed 3D visualization of asymmetric uptake in the affected condyle, with thresholds above 55% often indicating active disease [8]. Quantitative parameters, such as the condyle-to-clivus ratio, with a reported cutoff of >1.4, have demonstrated a specificity of 100% and a sensitivity of 85.7%, establishing SPECT/CT as a reliable diagnostic tool for active condylar growth [9]. SPECT/CT further outperforms planar imaging by detecting subtle asymmetries that planar techniques might overlook and by distinguishing condylar hyperplasia from other conditions, such as asymmetric mandibular hyperplasia [10]. This precision is essential for guiding treatment, as active growth often requires surgical intervention, while inactive cases may benefit from conservative approaches [8].

It is important to note that increased uptake on scintigraphy can also result from concurrent TMJ disorders or degenerative changes, which could mislead interpretation without a thorough clinical correlation. Consequently, integrating scintigraphy findings within the broader clinical and radiological context is essential to ensure accurate diagnosis and to guide effective treatment strategies.

Mandibular hypercondylia (HCM) must be differentiated from several similar conditions. Among these, osteochondroma, a benign, well-circumscribed tumor more commonly found in long bones [11], and fibrous dysplasia, characterized by the replacement of normal bone tissue with fibrous tissue leading to deformations. A CT scan is crucial for establishing the diagnosis. Finally, malignant bone tumors such as chondrosarcoma and osteosarcoma should be considered, although they are rare and typically associated with poorly defined bone destruction. Clinical evaluation, imaging studies (CT or MRI), and sometimes histopathological analysis are necessary to confirm the diagnosis [12].

Surgical treatment is often indicated when hypercondylia causes significant aesthetic and functional deformities. Surgical management depends on the growth activity of hypercondylia, the patient's age, and the impact on mandibular function and facial aesthetics. Surgical indications include active hypercondylia, confirmed by bone scintigraphy, indicating ongoing condylar growth that would worsen the asymmetry.

In this case, partial condylectomy can halt growth and stabilize the condition, improving both function and aesthetics. In cases of severe facial asymmetry with jaw deviation, orthognathic surgery may be required alongside condylectomy [13]. In the presence of malocclusion and TMJ pain or dysfunction associated with hypercondylia, due to the overload on the opposite TMJ, condylectomy can help relieve pain by eliminating the asymmetric growth [14].

In some cases, hypercondylia creates imbalances that complicate dental prosthesis placement. Surgical correction is necessary to stabilize the mandibular structure before complete dental rehabilitation, ensuring better prosthesis adaptation and stable occlusion.

In more complex cases, particularly when hypercondylia has caused severe malocclusion or significant facial asymmetry, bimaxillary orthognathic surgery may be indicated. This procedure involves mandibular osteotomy combined with maxillary osteotomy to reposition the upper and lower jaws simultaneously, restore functional occlusion, and restore facial symmetry. It is often the treatment of choice in patients with Class III malocclusions or significant facial deviations [14].

## Conclusion

Mandibular hypercondylia is a rare pathology that, due to the asymmetric growth of the mandibular condyle, can lead to significant functional, aesthetic, and psychological consequences. Diagnosis relies on a combination of clinical examinations and imaging techniques, including panoramic radiography, 3D CT scans, and planar or tomographic bone scintigraphy, which help assess condylar growth and activity. Treatment is often surgical, with condylectomy being the primary option to stabilize asymmetric growth, sometimes in combination with orthognathic surgery to correct major facial deviations and restore occlusion. Early and multidisciplinary management is crucial for optimizing aesthetic and functional outcomes and improving the patients' quality of life.

## Patient consent

Written informed consent was obtained from a legally authorized representative(s) for anonymized patient information to be pub- lished in this article.
